# Experimental Therapeutics for Challenging Clinical Care of a Patient with an Extremely Rare Homozygous *APOC2* Mutation

**DOI:** 10.1155/2020/1865489

**Published:** 2020-03-27

**Authors:** Masako Ueda, Anna Wolska, Frances M. Burke, Maria Escobar, Laura Walters, Dusanka Lalic, Robert A. Hegele, Alan T. Remaley, Daniel J. Rader, Richard L. Dunbar

**Affiliations:** ^1^Department of Medicine, University of Pennsylvania, Philadelphia, PA, USA; ^2^Lipoprotein Metabolism Laboratory, NHLBI, National Institutes of Health, Bethesda, MD, USA; ^3^Division of Cardiovascular Medicine, Department of Medicine, University of Pennsylvania Health System, Philadelphia, PA, USA; ^4^Department of Medicine, and Robarts Research Institute, Schulich School of Medicine and Dentistry, Western University, London, Ontario, Canada; ^5^Translational Medicine and Human Genetics, Department of Medicine, University of Pennsylvania, Philadelphia, PA, USA; ^6^Department of Medicine, Corporal Michael J. Crescenz VA Medical Center, Philadelphia, PA, USA; ^7^Amarin Pharmaceuticals, Bedminster, NJ, USA

## Abstract

**Background:**

Among many causes of hypertriglyceridemia (HTG), familial chylomicronemia syndrome (FCS) is a rare monogenic disorder that manifests as severe HTG and acute pancreatitis. Among the known causal genes for FCS, mutations in *APOC2* only account for <2% of cases. Medical nutrition therapy is critical for FCS because usual triglyceride- (TG-) lowering medications are ineffective. Therapeutic plasma exchange (TPE) with fresh frozen plasma (FFP) is an option to urgently reduce TG and pancreatitis episodes. Several novel biologics are under development to treat HTG and may provide therapeutic options for FCS in the future.

**Objective:**

We present the challenging care of a 43-year-old man with FCS with apoC-II deficiency and the results of two types of TPE and of investigational TG-lowering biologic therapies.

**Results:**

The patient's lipid profile was consistent with FCS. A novel homozygous variant was identified in *APOC2*, and its pathogenicity was confirmed. Even on a fat-restricted diet, his care was tremendously complicated with unremitting bouts of pancreatitis. TPE with FFP replacement lowered TG >90% post-sessions and appeared to reduce pancreatitis episodes. Experimental ANGPTL3 and *APOC3* inhibitors each lowered TG by >50%.

**Conclusions:**

Our case demonstrates the importance of delineating and defining the underlying etiology of a rare disorder to optimize therapy and to minimize unfavorable outcomes.

## 1. Introduction

Hypertriglyceridemia (HTG) has numerous etiologies. It is often polygenic and multifactorial [1]. However, familial chylomicronemia syndrome (FCS) or type I hyperlipoproteinemia with severe HTG (OMIM: 238600) is a rare and often underdiagnosed monogenic disorder, with an estimated prevalence of 1/1,000,000. There are five known causal genes for FCS: lipoprotein lipase (*LPL*), apolipoprotein (apo) C-II (*APOC2*), apoA-V (*APOA5*), lipase maturation factor 1 (*LMF1*), and glycosylphosphatidylinositol-anchored high-density lipoprotein-binding protein 1 (*GPIHBP1*). Each gene product supports proper LPL synthesis or its function, namely, TG hydrolysis [2].

Abdominal pain of varying intensity due to acute pancreatitis is often the first manifestation of FCS, typically presenting in childhood or adolescence. Unfortunately, it is often disregarded as a childhood illness or can prompt an unnecessary exploratory procedure. Therefore, arriving at the correct diagnosis is often delayed. Pancreatitis is the most serious, potentially fatal, consequence of FCS. Other reversible clinical features include dermatological eruptive xanthomas, ophthalmological lipemia retinalis, and hepatosplenomegaly; however, not all features are present in every patient.

Fasting chylomicronemic plasma appears milky and viscous with TG > 1,000 mg/dL (11.3 mmol/L). Three pretreatment lipid findings may help distinguish FCS (Table 1) from polygenic or multifactorial chylomicronemia (type V hyperlipoproteinemia) [3]: (1) TG/total cholesterol (TC) ratio >5 (mg/dL)/(mg/dL) or >2.2 (mmol/L)/(mmol/L), (2) TG/apoB ratio >8.8 (mg/dL)/(mg/dL) or ≥10 (mmol/L)/(g/L), and (3) low apoB <75 mg/dL (0.75 g/L). ApoB is typically high in polygenic chylomicronemia [4].

Historically, the diagnosis of FCS relied on LPL functional analysis, but this is not standardized and is only offered at a few specialized centers [2]. Despite recent development of the FCS scoring system using clinical information to delineate FCS [5], definitive diagnosis by clinical criteria alone is still challenging due to overlapping features among chylomicronemias [1, 6, 7]. Nevertheless, characteristic clinical features and FCS scoring may be valuable in screening for FCS. Ultimately, in order to definitively diagnose FCS, molecular testing targeting FCS-associated genes is necessary and may be practical as genetic sequencing becomes more accessible due to declining cost and expanding availability [8, 9].

The most effective treatment for FCS is medical nutrition therapy with a fat-restricted diet with <15% of total calories as fats. FDA-approved TG-lowering medications are only marginally effective for FCS because they predominantly affect very-low-density lipoprotein (VLDL) metabolism more than the chylomicron metabolism and require “functional” LPL. Unresponsiveness to TG-lowering drugs could be an important clue to the diagnosis of FCS. Regardless, maintaining a long-term fat-restriction is very difficult.

Besides dietary management, therapeutic plasma exchange (TPE) with fresh frozen plasma (FFP) is sometimes used urgently to alleviate severe HTG and may curtail recurrent pancreatitis although it is cubersome chronically [10]. Several novel biologics suppressing the function of physiologic LPL inhibitors, such as angiopoietin-like 3 (ANGPTL3) and apoC-III, are under clinical development to treat HTG and may become treatment options for FCS in the future.

## 2. Objective

We present the challenging care of a 43-year-old man with FCS with a novel homozygous *APOC2* mutation and the effects of two types of TPE and of investigational TG-lowering biologics.

## 3. Method

The patient participated in an IRB-approved study investigating rare lipid disorders at the University of Pennsylvania and provided his consent for medical record review.

## 4. Case Presentation

A 43-year-old-man, originally from Sudan, presented to the Lipid Clinic, after being diagnosed with HTG during his first episode of pancreatitis at age 30. He had no physical stigmata of FCS. His fasting plasma appeared milky and turbid. His highest reported TG level was 7,112 mg/dL (80.3 mmol/L), TC 455 mg/dL (11.8 mmol/L), and high-density lipoprotein cholesterol (HDL-C) 12 mg/dL (0.31 mmol/L). His TG/TC ratio of 15.6 (mg/dL)/(mg/dL) (6.81 (mmol/L)/(mmol/L)) and profoundly low apoB of 13 mg/dL (<75 mg/dL) or 0.13 g/L, as well as TG/apoB ratio of 547 (mg/dL)/(mg/dL), 617 (mmol/L)/(g/L), were consistent with FCS. In addition, his FCS score of 10 provided further evidence that he was likely to have FCS [7].

**Figure 1:**
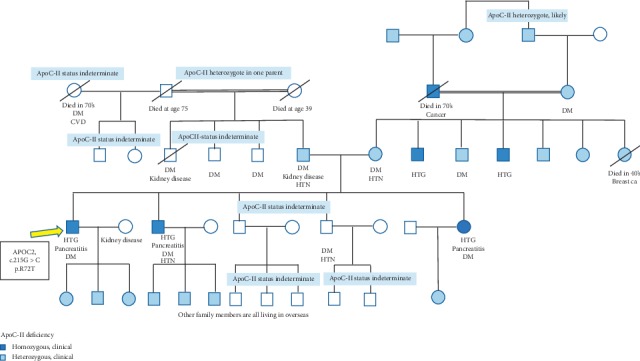
Patient's family pedigree. The patient is originally from Sudan, and many family members live overseas. Consanguinity is present on both sides. The patient's paternal and maternal grandparents are distantly related. The patient's sister and one brother have similar medical history with HTG and pancreatitis, as well as DM. His maternal uncles have HTG, and his maternal grandfather was known to have had HTG.

Around the same time, he was also diagnosed with diabetes mellitus (DM), independent of pancreatitis. His family history was very suspicious for FCS with consanguinity on both sides of the family. Notably, his sister and one brother, as well as two maternal uncles had HTG with or without pancreatitis (Figure 1).

We identified a novel homozygous *APOC2* variant, c.215 G > C, p.R72 T [8], and proved its pathogenicity by demonstrating restored lipolytic activity after adding an apoC-II mimetic peptide (C-II-a) to the patient's plasma, reported elsewhere [11]. The variant, located in an amphipathic helical region of the protein, is predicted to disrupt its lipid-binding ability [12]. Therefore, we have definitively diagnosed the patient with FCS due to very rare apoC-II deficiency as an adult.

### 4.1. Management Challenges

His clinical course has been tumultuous with recurrent bouts of pancreatitis, ultimately developing chronic pancreatitis. Moreover, he suffered what appeared to be a transient ischemic attack (TIA) during a clinic visit. He became confused and could not stay standing. Fortunately, with fluid resuscitation, he recovered without residual sequelae. Hyperviscosity and its associated neurological deficits due to extremely high TG levels have been reported [13]. We suspected that plasma hyperviscosity resulting from dehydration after a prolonged travel excursion with the underlying FCS itself was likely to have contributed to this event.

Most of all, his dietary management proved problematic. In addition to a fat-restricted diet, limiting carbohydrate intake was required for his DM. Consequently, protein intake had to be maximized to fulfill his caloric needs. The patient often became confused about his dietary regimen, receiving conflicting advice from various healthcare providers who were unfamiliar with FCS. This did not improve until our nutritionist conferred with his other nutritionist to unify his management plan.

### 4.2. Chronological Report of Experimental Therapeutics

**Figure 2:**
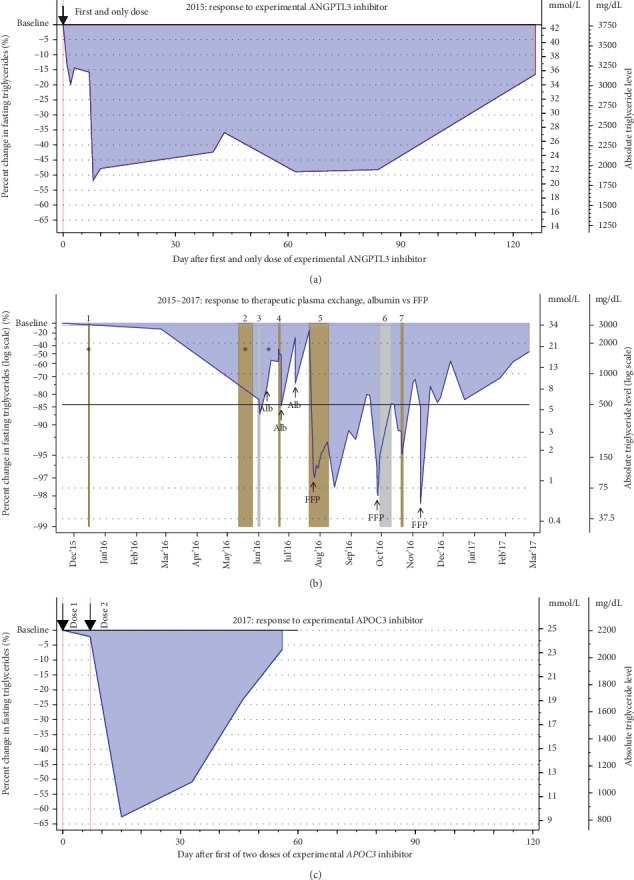
Fasting TG response to chronological therapeutic attempts. (a) The line represents our patient's TG response to a single dose of an experimental ANGPTL3 inhibitor. Within 10 days post-dose, TG fell >50%, remaining –50% to –35% below baseline for about 90 days afterwards. Overall, this effect lasted for about 120 days. (b) The line represents the patient's fasting TG in response to therapeutic plasma exchange (TPE) using two types of replacement infusions: albumin and fresh frozen plasma (FFP). The asterisks (*∗*) indicate times when TG data are unavailable. The vertical bars indicate 7 hospital admissions: admissions 1, 2, 4, 5, and 7 (brown) for pancreatitis, admission 3 (gray) for anorexia, weakness, and anemia, admission 6 (gray) for the management of duodenal obstruction for pancreatic cysts, receiving total parental nutrition before converting to jejunostomy feeding followed by slowly resuming oral intake after discharge. 5% albumin replacement only briefly lowered TG to about 500 to 1000 mg/dL after exchanging 1.0 plasma volume (PV). After switching, three sessions with FFP replacement were more effective in TG-lowering, with a sharp drop of TG to <150 mg/dL immediately post-infusion 1 and to <75 mg/dL post-infusions 2 and 3. His TG remained <1000 mg/dL for about a month even after the discharge from the hospital. (c) The line presents our patient's TG response to two doses of an experimental APOC3 inhibitor that were given a week apart. His baseline TG of 2,200 mg/dL fell >60% within about 15 days of the first dose. TG remained <1,000 mg/dL (<50%) until past 30 days, and <2,000 mg/dL for 55 days post-dose 1.

In early 2015, he participated in an IRB-approved TG-lowering study, testing the effectiveness of an experimental ANGPTL3 inhibitor. After a single dose, his TG fell ∼50% from 3,437 mg/dL within 10 days (Figure 2(a)). The average reduction was ∼40% during the first 80 days, and TG remained below baseline for 90 days post-dose.

Unfortunately, his unstable medical status with recurrent bouts of pancreatitis precluded him from participating in another therapy at that time. He had 7 hospital admissions during one year, of which 6 occurred within a 6-month period. Five hospitalizations were for acute pancreatitis, and two were for related complications: anorexia, anemia, and surgical intervention to relieve an intestinal obstruction caused by chronic pancreatitis. By this time, the majority of pancreas was replaced by multiloculated, fluid-filled cysts, compromising both exocrine and endocrine function and worsening his DM.

During the latter half of 2016, we prescribed TPE sessions to curtail pancreatitis episodes. Reportedly, his sister and brother often relied successfully on TPE to limit their recurrent pancreatitis. Initially, we prescribed TPE of 1 plasma volume (PV) exchange with FFP every few weeks. However, the patient received albumin replacements for the first three sessions as per their facility protocol. On albumin TPE, TG fell 70–85% to <1,000 mg/dL but rebounded to >2,000 mg/dL within 2 weeks of each session (Figure 2(b)). On FFP replacement, as originally planned, TG initially fell >95% to <150 mg/dL and even to <75 mg/dL post-sessions 2 and 3. Moreover, TG-rebound was gradual, maintaining <1,000 mg/dL between sessions and even one month after discharge from the hospital.

In late 2016, the patient elected to discontinue TPE in order to participate in another IRB-approved study of an experimental *APOC3* inhibitor. He received two doses of the biologic a week apart in 2017. About a week after the second dose, his TG fell by >60% and remained <1,000 mg/dL for 40 days post-dose 1. TGs did not rebound back to >2,000 mg/dL until 50 days post-dose (Figure 2(c)). Overall, these experiences with novel biologics were quite promising.

## 5. Discussion

Severe HTG is vastly heterogeneous, and identifying the underlying cause can reveal the most optimal therapy [14]. Once FCS is diagnosed, a low-fat diet should be prescribed as first-line treatment, and TG-lowering medications can be prescribed as adjuncts.

Compared to usual patients with FCS, our patient's management was tremendously challenging because of his concurrent DM. Moreover, other healthcare providers were not well-versed on the management principles of apoC-II deficiency. Unfortunately, reliance on their experience in treating typical HTG patients became a barrier for our patient's care, and we suspect that this practice has exacerbated our patient's clinical course.

Successful use of TPE for extreme HTG has been reported previously [10]. In one study, patients with medication-refractory HTG at risk for pancreatitis underwent TPE with albumin replacement. The average TG level of 1,929 mg/dL pre-TPE was reduced to 762 mg/dL post-TPE, ∼60% reduction (*p* ≤ 0.0001). Importantly, TPE was effective in preventing recurrent pancreatitis. We predicted that TPE with FFP replacement, which contains exogenous apoC-II, would outperform albumin replacement in our patient with apoC-II deficiency by supplying the very protein our patient lacked, and the results concurred with this expectation. Our results indicate that TPE with FFP can be used as a last resort when apoC-II deficiency has been diagnosed.

Frustratingly, recently developed TG-lowering medications are still not suitable for FCS. Novel omega-3s, which promise to improve dyslipidemia and even to improve cardiovascular outcomes [15, 16], newer formulations of fibrates [17], and niacin [18] are all not expected to have an appreciable TG-lowering effect in FCS. Off-label use of a novel microsomal triglyceride transfer protein inhibitor may lower TGs, but an unfavorable safety and drug interaction profile would preclude its long-term use [19]. Considering this bleak outlook, our patient's responses to both ANGPTL3 and *APOC3* biologic inhibitors were especially encouraging. In theory, enhancing residual LPL function is the main mechanism of TG-lowering, achieved by the suppression of ANGPTL3 protein or *APOC3* translation, thereby alleviating LPL inhibition. However, it was intriguing that inhibiting ANGPTL3 also had a positive effect in our patient who had no detectably functional apoC-II, the key cofactor for LPL function [11]. Therefore, the full scope of the effects of ANGPTL3 inhibition may still be unknown. We hope that such biologics would become available in the near future for treatment of FCS.

Lastly, apoC-II mimetic peptides effectively lowered TG in preclinical animal models of apoC-II deficiency [20]. In fact, adding one of these peptides normalized LPL activity in an *ex vivo* assay of our patient [11]. Ultimately, an apoC-II mimetic may become the most specific therapeutic option for apoC-II deficiency.

## 6. Conclusion

Our patient's case clearly illustrates the importance of carefully delineating the cause of a rare condition to manage appropriately and to minimize unfavorable outcomes. More importantly, all healthcare providers should be especially vigilant when caring for a patient with an unfamiliar disorder.

## Figures and Tables

**Table 1:** Typical characteristics of familial chylomicronemia syndrome.

General features of FCS	

Electrophoretic characteristic	Type I hyperlipoproteinemia
Genetics	Monogenic (biallelic, autosomal recessive)
Prevalence	1 in 100,000 to 1,000,000
Disease onset	Childhood/adolescent > adulthood

Clinical manifestations	Abdominal pain
	Eruptive xanthoma
	Lipemia retinalis
	Pancreatitis
	Hepatosplenomegaly
	Lactescent plasma

Response to TG-lowering medications	None to marginal

Association with CVD	None to minimal risk

Lipoprotein features of FCS	
Major lipoproteins	Chylomicrons
Lipoprotein characteristics^1,2^	TG: >1,000 mg/dL; >11.3 mmol/L
	TG/TC ratio:
	>5 (mg/dL)/(mg/dL); >2 (mmol/L)/(mmol/L)
	TG/ApoB ratio:
	≥8.8 (mg/dL)/(mg/dL); ≥10 (mmol/L)/(g/L)
	ApoB: <75 mg/dL; <0.75 g/L

FCS: familial chylomicronemia syndrome, CVD: cardiovascular disease, TG: triglyceride, TC: total cholesterol, and ApoB: apolipoprotein B.
